# Offer of integrative and complementary health practices for the elderly in health services: A protocol for systematic review and meta analysis

**DOI:** 10.1097/MD.0000000000032856

**Published:** 2023-02-17

**Authors:** Nathália Priscilla Medeiros Costa Diniz, José Felipe Costa da Silva, Ana Tânia Lopes Sampaio, Gilson de Vasconcelos Torres, Mariana Cabral Schveitzer, Thaiza Teixeira Xavier Nobre

**Affiliations:** a Graduate Program in Collective Health, Federal University of Rio Grande do Norte, Natal/RN, Brazil; b Graduate Program in Health Sciences, Federal University of Rio Grande do Norte, Natal/RN, Brazil; c Department of Collective Health, Federal University of Rio Grande do Norte, Natal, RN, Brazil; d Department of Nursing, Federal University of Rio Grande do Norte, Natal, RN, Brazil; e Department of Preventive Medicine, Federal University of São Paulo, São Paulo, SP, Brazil; f Faculty of Health Sciences of Trairi, Federal University of Rio Grande do Norte, Santa Cruz-RN, Brazil.

**Keywords:** complementary therapies, health of the elderly, health services for the elderly, protocol, systematic review

## Abstract

Integrative and complementary health practices (ICHP) question the hegemony of the biomedical, technical and hospital paradigm, as they are an important axis in the process of redefining the health care model. Understanding how ICHP are offered to the elderly population can help to improve the production of changes in care and in the daily life of health services.

**Objective:**

To identify and summarize the scientific evidence on the provision of ICHP for the elderly in health services.

**Methods:**

This is a research protocol for a scoping review following the recommendations of the Extension for Scoping Reviews method proposed by the Joanna Briggs Institute. Studies will be collected in the following databases, Latin American and Caribbean Health Sciences Literature, Web of Science, Scopus, Scielo, Online System for Searching and Analyzing Medical Literature (MEDLINE), Embase, Virtual Library in Health and gray literature. Two independent reviewers will perform screening, data extraction, and risk of bias assessment using the Joanna Briggs Institute Critical Assessment Checklist. For the quality of evidence, the Grading of Recommendations, Assessment, Development and Evaluation analysis will be used.

**Results:**

This review will provide information on the provision of ICHP for the elderly population in health services.

**Conclusions:**

This scoping review will provide evidence to help health professionals, managers and users to recognize more effective therapeutic inventions for promoting, preventing and protecting comprehensive health at different levels of care.

## 1. Introduction

Due to the demographic transition that is taking place in the Brazilian population, there is a need to promote and create policies aimed at encouraging the individual and collective well-being of the elderly population. The implementation of mechanisms capable of reducing the psychic, social and physical impacts that are present in the lives of the elderly and those in the process of transition from youth to old age is necessary. Given this scenario, we understand that this population needs more specific attention focused on prevention, promotion and rehabilitation for their senility and/or senescence process.^[1]^

The Integrative and Complementary Health Practices (ICHP) are based on the practice of care, knowledge and products for therapeutic use that do not belong to conventional or allopathic medicine, whose treatment aims to induce a natural state of harmony and balance in the entire organism. The World Health Organization (WHO) calls the field of Integrative Practices Traditional Medicine and the field of complementary practices Complementary/Alternative Medicine (TM/CAM). Since the 1970s, the WHO has encouraged Member States to formulate and implement public policies for the rational and integrated use of TM/CAM in Primary Health Care.^[2]^

From this perspective, in 2006, the Brazilian Ministry of Health published the National Policy on Integrative and Complementary Health Practices in the Unified Health System (NPICHP/SUS).^[3]^ An achievement that began in a procedural way, gaining legitimacy and being one of the recommendations of the World Health Organization (OMS).^[4]^

The NPICHP initially inserted five ICHP in the list of procedures, in 2017 and 2018 another 24 ICHP were inserted in the National List of Health Actions and Services (RENASES), totaling 29. This means that, in Brazil, the population has the right to opt for these differentiated comprehensive care.^[3]^

The ICHP offer possibilities of experiences that prevent imbalance and complement the allopathic treatment when the person is already sick, expanding the possibility of improvement/cure and integrate the different dimensions of the being, promoting well-being, raising self-esteem, increasing immunity, increasing the production of endorphins, serotonin, favoring states of happiness.^[5]^

Acting with the ICHP means going far beyond physical therapeutic care with the disease, it means looking at and focusing on health, on the person, working with human energy, with the determinants of the health process, illness, with the singularities, with the vibrational field of the subject determined by his/her history and life condition. The symptom should be seen as a sign that something is wrong and investigate the cause, seek to identify and resolve the factor generating that imbalance. The investment should be in health promotion and disease prevention, aiming to provide self-knowledge, empowerment, self-care and expansion of the state of consciousness.^[6]^

In addition, trust in health care, both in terms of the service and the professional, is relevant for the patients’ willingness to use complementary medicine and to prevent ICP from being chosen without guidance,^[7,8]^ highlighting the need to investigate the demand for ICHP as a way to guarantee the effectiveness, safety and quality of these practices, in addition to promoting access and rational use, as recommended by the WHO.^[2,8,9]^

It is important to emphasize that the implementation of the NPICHP had a political, technical, economic, social and cultural character, since it establishes national guidelines for the use of ICHP, based on experiences and practices already adopted in health services that obtained satisfactory results. This fact further facilitated the dissemination of these practices in various parts of the country and the world.^[10]^

Based on this, Brazil has stood out as one of the 69 Member States of the WHO that have specific policies and strategies for the use of ICHP.^[2]^ After the creation of the NPICHP, 30% of Brazilian municipalities adopted their own regulations for the use of these therapies, indicating a significant increase in practices in health care; and Primary Health Care is one of the main environments for its application.^[11]^ In this context, this review is justified when trying to understand the current supply/use scenario of ICHP in health services.

A preliminary search was performed on PROSPERO and no current or ongoing scoping reviews or systematic reviews on the topic were identified. Thus, the objective of this protocol is to map the available evidence on the offer of ICHP for elderly people in health services.

### 1.1. Research question

How are ICHPs offered to the elderly in health services?

### 1.2. Inclusion criteria

#### 1.1.2. Participants.

Studies carried out with elderly people aged 60 or over, without sex/gender restrictions, will be included.

#### 2.1.2. Concept.

This review will consider studies that explore the offer of ICHP and describe their strategies and/or approaches with health services. As well as describing the positive impacts on the offer of ICHP for the health of the elderly.

#### 3.1.2. Context.

The present review will mainly consider studies focusing primarily on the scope of health services, not limited to these aspects, as well as any geographic region.

#### 4.1.2. Types of studies.

This scoping review will consider quantitative, qualitative, and mixed methods study designs for inclusion published in full in English, Spanish, and Portuguese from 2006 to 2022. In addition, systematic reviews and text and opinion articles will be considered for inclusion in the proposal scope review.

## 2. Method

This is a research protocol for a scoping review following the recommendations of the Extension for Scoping Reviews (PRISMAScR)^[12]^ method proposed by the Joanna Briggs Institute.^[13]^ The protocol for this study was registered in the Open Science Framework (https://osf.io/4NXUK/).^[12]^

### 2.1. Research strategies

The search strategy will aim to locate published and unpublished primary studies, reviews, and text and opinion articles. A limited initial search in the Virtual Health Library (via capes journals), Embase, Medline and CINAHL was performed to identify articles on the subject. The text words contained in the titles and abstracts of relevant articles and the indexing terms used to describe the articles were used to develop a complete search strategy (see Appendix I, http://links.lww.com/MD/I430 on Supplemental Digital Content). The search strategy, including all identified keywords and indexing terms, will be adapted for each information source included. Reference lists of articles selected for full-text review included in the review will be selected as additional articles.

### 2.2. Databases to be searched include

● MEDLINE (Ovid Online - www.ovid.com);
● EMBASE (Ovid Online - www.ovid.com);
● PubMed (www.ncbi.nlm.nih.gov/pubmed/);
● LILACS (lilacs.bvsalud.org/);
● SCIELO (www.scielo.org/php/index.php);
● CINAHL (www.ebscohost.com);
● SCOPUS (via capes journals);
● WEB OF SCIENCE (via capes journals);
● EMBASE (Ovid Online - www.ovid.com);
● Virtual Health Library (via capes journals).
● Sources of unpublished studies and gray literature to be researched include Catalog of Theses and Dissertations of Brazil;
● Portuguese Open Access Scientific Repository;
● Gray Literature Information System in Europe (Open Grey).

### 2.3. Selection of studies

After the bibliographic search, all identified records will be exported from the databases in EndNote, Export, Refman/RIS or Text format and included in the Rayyan QCRI^[14]^ platform. This platform was developed by the Qatar Computing Research Institute, available free of charge and online, offering a wide range of resources, including uploading citations in different formats, navigation, automatic extractions to exclude duplicate citations. For this study the Rayyan QCRI will be used for exclusion of duplicates and required for title and abstract analysis. After a pilot test, titles and abstracts will be selected by two independent reviewers for evaluation according to the review’s inclusion and exclusion criteria.

The full text of selected citations will be evaluated in detail according to the inclusion criteria, by two independent reviewers. The reasons for excluding full-text articles that do not meet the inclusion criteria will be recorded and reported in the scoping review. Any disagreements that arise between reviewers at each stage of the selection process will be resolved by discussion or with a third reviewer. Research results will be reported in full in the final scope review and presented in a PRISMA flowchart (see Appendix II, http://links.lww.com/MD/I431 on Supplemental Digital Content).^[15]^

### 2.4. Data extraction

Data will be extracted from the articles included in the scoping review by two independent reviewers using two data extraction tools, developed by the reviewers in Excel spreadsheet format. The data extracted in the first tool will include specific details about the identification of the publication containing the author/year information; country of study, language; magazine; study design and participants; the second worksheet will present details of ICHP offers to the elderly with the type of intervention; illnesses; place of offers; target audience; type of ICHP, number of procedures and conclusion. This extraction will be carried out by the two reviewers and then compared, if there is no agreement, the third reviewer will be activated. The draft data extraction tool will be modified and revised as needed during the data extraction process for each included article. Modifications will be detailed in the full scope review.

A draft extraction tool is provided (see Appendix III, http://links.lww.com/MD/I432 on Supplemental Digital Content).

### 2.5. Data analysis and presentation

Data will be descriptively mapped and presented through tables and figures with the distribution of articles by period of publication, country, and main variables found. A narrative summary of all results will accompany the tables and figures that describe the main strategies for offering ICHP to the elderly population in health services.

### 2.6. Ethics and dissemination

For the development of this study, approval of ethics and consent is not necessary because it is a systematic review that will use secondary studies.

### 2.7. Access and funding statement

The research was funded by the Federal University of Rio Grande do Norte, through the Postgraduate Pro-rectory, and the Postgraduate Program in Health Sciences. Data sharing not applicable to this article as no datasets were generated or analyzed during the current study.

## 3. Discussion

Studies^[16]^ point to the good results obtained with the insertion of ICHP in the care of the elderly in Brazil, referring to the high level of satisfaction of the elderly when experiencing these practices. They also point out the cost-effectiveness of these actions, since most of them are light technologies, using natural external practices, which do not require greater technological devices.

However, both in Brazil^[17]^ and in China,^[18]^ public policies aimed at the health of the elderly need greater increment, involvement and participation of a series of subjects: those directly interested—the elderly themselves—managers, family members and health professionals.

With that in mind, to put into practice all the necessary actions for healthy aging and quality of life, it is necessary to rethink and redesign care for the elderly, focusing on these individuals and their particularities. This will bring benefits not only to the elderly, but also quality and sustainability to the Brazilian health system.^[19]^ It should be considered that comprehensive care is the result of the ways in which the different practices of workers in health care for the elderly are articulated.^[6]^

It is expected that, at the conclusion of this review, the main findings may contribute to enhancing the offer of ICHP for the elderly, in the various health services with a view to improving knowledge and dissemination of the existence of different therapies aimed at comprehensive health care and subsidies to develop them in a broader way, in daily practice.

## Author contributions

**Conceptualization:** Nathália Priscilla Medeiros Costa Diniz, José Felipe Costa da Silva.

**Methodology:** Mariana Cabral Schveitzer.

**Project administration:** Thaiza Teixeira Xavier Nobre.

**Supervision:** Ana Tânia Lopes Sampaio.

**Writing – original draft:** Gilson de Vasconcelos Torres.

**Writing – review & editing:** Nathália Priscilla Medeiros Costa Diniz, José Felipe Costa da Silva.

## Supplementary Material
