# Supplementary material for: Offer of integrative and complementary health practices for the elderly in health services: A protocol for systematic review and meta analysis
Source: Medicine (Baltimore). 2023 Feb 17;102(7):e32856. doi: 10.1097/MD.0000000000032856 (PMC9936020; doi:10.1097/MD.0000000000032856)
Supplement: Supplementary file 1 [file medi-102-e32856-s001.pdf]

Appendix I: Virtual Health Library (via capes journals), EMBASE and CINAHL. Research conducted in December 2022.

**Search**	**Query**	**Records retrieved**	**Base**	**Observation**
#1	((("Serviços de Saúde para Idosos" OR "Health Services for the Aged" OR "Servicios de Salud para Ancianos" OR "Asistencia a los Ancianos" OR "Old Age Assistance" OR "assistência a Idosos" OR "Salud del Anciano" OR "Health of the Elderly" OR "Saúde do Idoso")) OR (ti:(idoso* OR "adulto mayor" OR anciano* OR aged)) OR (ti:("Pessoa Idosa" OR "Pessoa de Idade" OR "Pessoas Idosas" OR "Pessoas de Idade" OR "População Idosa" OR "Persona Mayor" OR "Persona de Edad" OR "Personas Mayores" OR "Personas de Edad" OR elderly))) AND (("Terapias Complementares" OR "Complementary Therapies" OR "Terapias Complementarias" OR "complementary therapy" OR "Práticas Complementares e Integrativas" OR "Práticas Integrativas e Complementares" OR "Práticas de Saúde Complementares e Integrativas" OR "Práticas de Saúde Integrativas e Complementares" OR "Terapias Complementares e Integrativas" OR "Tratamentos Complementares" OR "Prácticas Complementarias e Integradoras" OR "Prácticas Integradoras y Complementarias" OR "Prácticas de Salud Complementarias e Integradoras" OR "Prácticas de Salud Integradoras y Complementarias") OR (ti:("Terapias Alternativas" OR "Terapia Alternativa" OR "alternative therapy" OR "alternatives therapies")) OR (ti:((complementar* OR integrativ* OR integradora* OR traditional* OR tradiciona*)))) AND NOT (db:"MEDLINE") AND (fulltext:("1") AND la:("pt" OR "en" OR "es")) AND (year_cluster:[2006 TO 2022])	503	BVS (excluding MEDLINE)	

#2	("Health Services for the Aged" OR "Old Age Assistance" OR "Health of the Elderly") AND ("Complementary Therapies")	15	MEDLINE	
#3	("Health Services for the Aged" OR "Old Age Assistance" OR "Health of the Elderly") AND "Complementary Therapies"	30	CINAHL	
#4	('health services for the aged'/exp OR 'health services for the aged' OR 'old age assistance'/exp OR 'old age assistance' OR 'health of the elderly') AND 'complementary therapies' AND ([english]/lim OR [portuguese]/lim OR [spanish]/lim) AND ([embase]/lim OR [pubmed-not-medline]/lim) AND [2017-2022]/py	6	EMBASE	
#5	#1 AND #2 AND #3 AND #4	584		
Language limits: English, Portuguese and Spanish.<br>Publication date: 2006 to 2022.<br>Geographic area: without delimitation.				
