# Supplementary material for: Offer of integrative and complementary health practices for the elderly in health services: A protocol for systematic review and meta analysis
Source: Medicine (Baltimore). 2023 Feb 17;102(7):e32856. doi: 10.1097/MD.0000000000032856 (PMC9936020; doi:10.1097/MD.0000000000032856)
Supplement: Supplementary file 2 [file medi-102-e32856-s002.pdf]

Appendix II: PRISMA 2020 flow diagram for systematic review and meta analysis which included searches of databases and registers only.

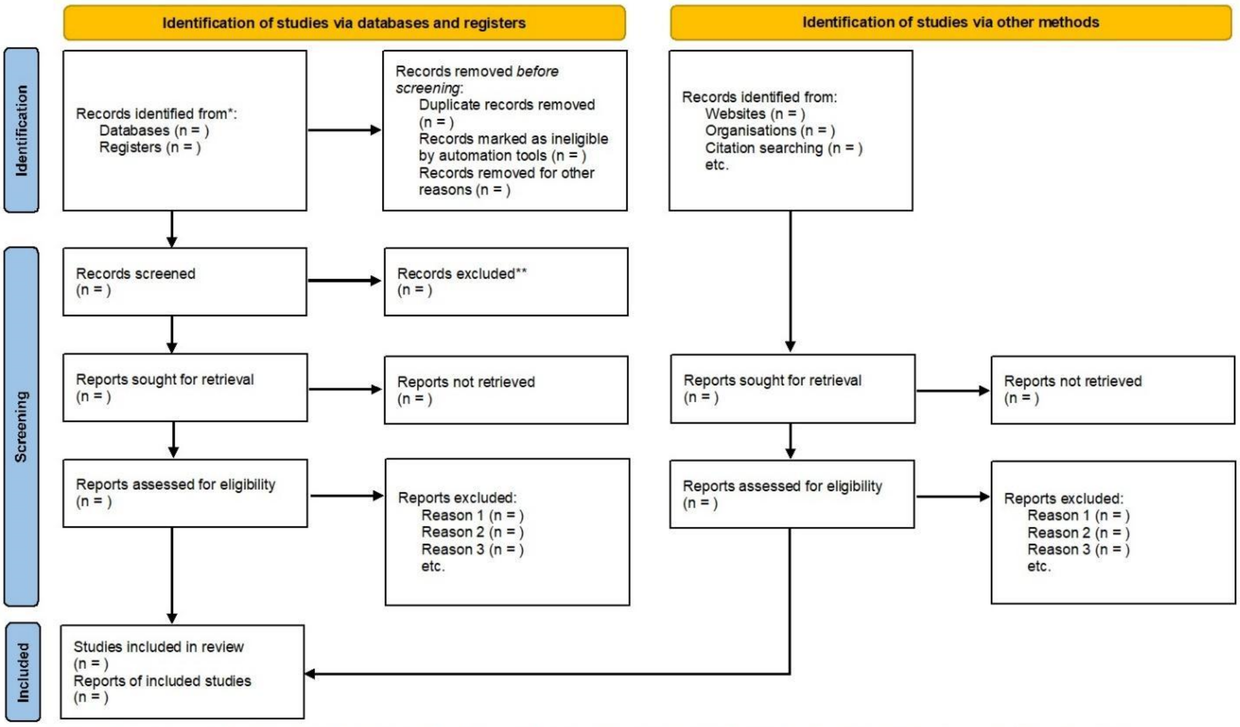

*Consider, if feasible to do so, reporting the number of records identified from each database or register searched (rather than the total number across all databases/registers).
**If automation tools were used, indicate how many records were excluded by a human and how many were excluded by automation tools.

From: Page MJ, McKenzie JE, Bossuyt PM, Boutron I, Hoffmann TC, Mulrow CD, et al. The PRISMA 2020 statement: an updated guideline for reporting systematic reviews. BMJ 2021;372:n71. doi: 10.1136/bmj.n71. For more information, visit: http://www.prisma-statement.org/
