# Supplementary material for: Offer of integrative and complementary health practices for the elderly in health services: A protocol for systematic review and meta analysis
Source: Medicine (Baltimore). 2023 Feb 17;102(7):e32856. doi: 10.1097/MD.0000000000032856 (PMC9936020; doi:10.1097/MD.0000000000032856)
Supplement: Supplementary file 3 [file medi-102-e32856-s003.pdf]

Appendix III: Data extraction instrument

Author/ Year	Country	Language	Journal	Study design	Population	Intervention	Effects	ICHP type	Number of Procedures	Health conditions	Intervention local	Conclusion
